# The Role of the Mammalian Target of Rapamycin in Microglial Phenotypic Polarization

**DOI:** 10.3390/cells15181654

**Published:** 2026-09-14

**Authors:** Kaitlyn J. Partridge, Allison D. Ebert

**Affiliations:** 1 Department of Cell Biology, Neurobiology, and Anatomy, Medical College of Wisconsin, 8701 West Watertown Plank Road, Milwaukee, WI 53226, USA; kpartridge@mcw.edu; 2 Neuroscience Research Center, Medical College of Wisconsin, 8701 West Watertown Plank Road, Milwaukee, WI 53226, USA

**Keywords:** microglia, mammalian target of rapamycin, neuroinflammation

## Abstract

**Highlights:**

**What are the main findings?**

- mTOR signaling regulates microglial phenotypic plasticity by coordinating many cellular processes including metabolic reprogramming, inflammatory mediator production, and cytoskeletal remodeling.
- mTORC1 generally amplifies pro-inflammatory microglial responses, whereas mTORC2 appears to promote anti-inflammatory programs in a context-dependent manner.

**What are the implications of the main findings?**

- The coordination of mTORC1 and mTORC2 signaling may determine microglial states.
- mTOR modulation may be a therapeutic target for neuroinflammatory diseases.

**Abstract:**

Microglia are adaptive immune cells that maintain central nervous system homeostasis and respond dynamically to injury, infection, and other neurological insults. While traditionally classified into resting, pro-inflammatory “M1”, and anti-inflammatory “M2” states, advances in multi-omic profiling technologies have established that microglial phenotypes exist along a multidimensional and context-dependent continuum. The mammalian target of rapamycin (mTOR), a central regulator of cellular metabolism, growth, survival, and protein synthesis, has emerged as a potential central mediator of these state transitions through distinct activities downstream of mTOR complex 1 (mTORC1) and mTOR complex 2 (mTORC2). In this review, we examine current evidence linking mTOR signaling to microglial phenotypic polarization and functional plasticity. Generally, evidence suggests that mTORC1 acts as a context-dependent amplifier of inflammatory responses, whereas mTORC2 promotes anti-inflammatory and neuroprotective programs; however, the effects of either complex vary according to disease context. Understanding the balance and coordination of mTORC1 and mTORC2 signaling programs may clarify mechanisms that underly chronic neuroinflammation and guide the development of targeted therapies for neuroinflammatory disorders including Alzheimer’s disease, stroke, and epilepsy.

## 1. Introduction

Microglia are highly specialized immune cells that serve as the primary sentinels of the central nervous system (CNS), continuously monitoring the neural environment to preserve homeostasis and coordinate responses to injury, infection, and neurodegeneration [1]. Their phenotypic plasticity enables them to rapidly transition between diverse functional states in response to environmental cues, allowing them to regulate processes ranging from synaptic remodeling and debris clearance to inflammatory signaling and tissue repair [2]. These responses have historically been categorized into resting (M0), classically activated (M1), and alternatively activated (M2) [1,3]. However, advances in profiling technologies have revealed that microglial phenotypes exist along a multidimensional and highly dynamic continuum rather than within binary states [2]. This new information has placed importance on understanding the molecular mechanisms that govern microglial state transitions and their implications within neuroinflammatory diseases.

**Figure 1:**
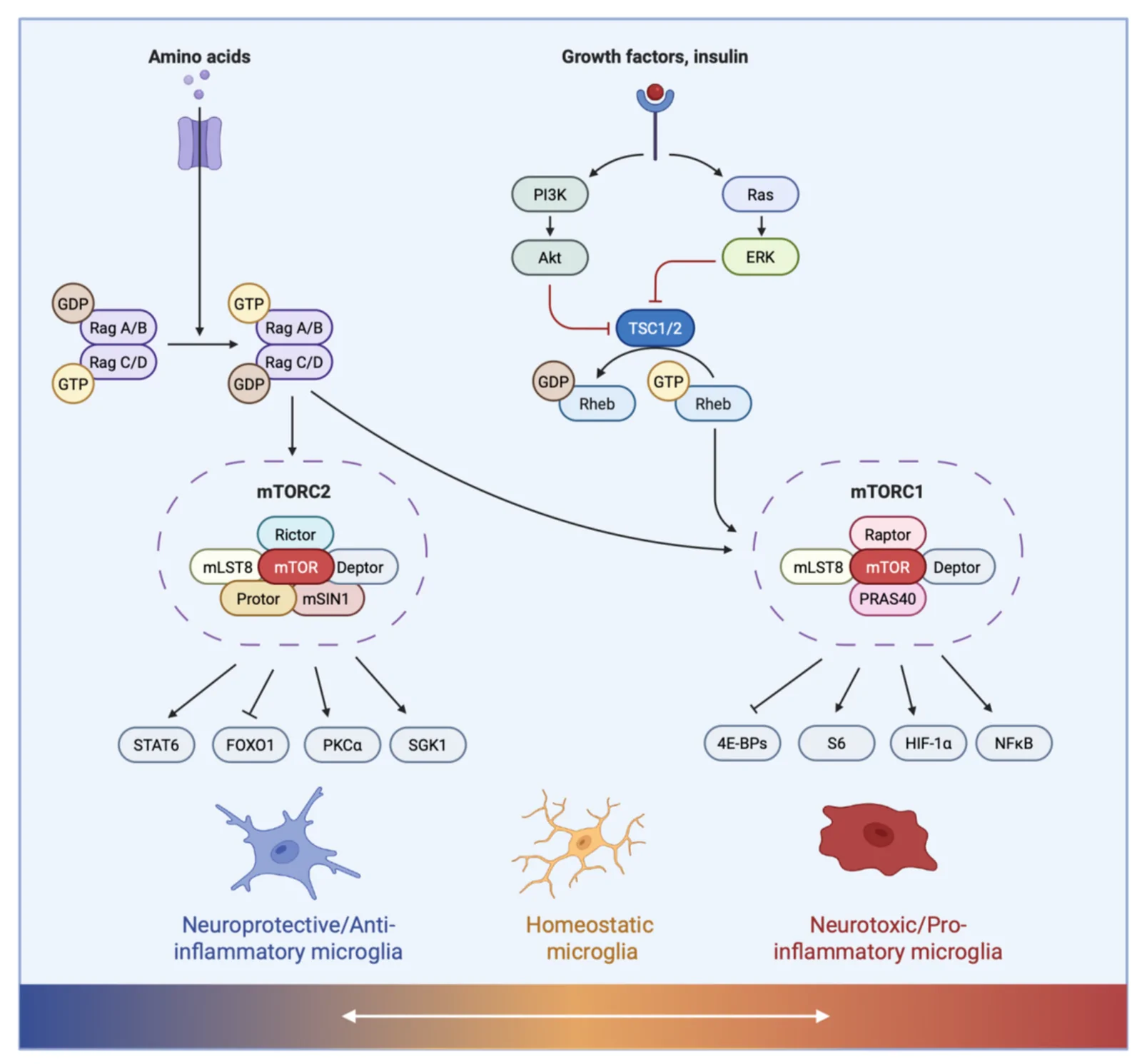
Proposed model of mTOR-dependent microglial state regulation. Amino acids regulate Rag GTPases, promoting mTOR complex signaling, whereas growth factors and insulin stimulate the PI3K-Akt and Ras-ERK pathways. Akt and ERK phosphorylate and inhibit the TSC1/2 complex, permitting RHEB-GTP-dependent mTORC1 activation. mTORC1, comprising mTOR, Raptor, Deptor, mLST8, and PRAS40, regulates downstream effectors including the 4E-BPs, S6K, HIF-1α, and NFκB and is associated with a neurotoxic or pro-inflammatory microglial phenotype. mTORC2 comprises mTOR, Rictor, mSIN1, Protor, mLST8, and Deptor. It modulates downstream effectors such as STAT6, FOXO1, PKCα, and SGK1 and is associated with promoting a neuroprotective or anti-inflammatory microglial phenotype. Taken together, this model depicts nutrient- and growth factor-responsive mTOR signaling as a potential determinant of microglial states or phenotypes along a continuum, ranging from neuroprotective to homeostatic to neurotoxic. Created in BioRender. Partridge, K. (2026) https://BioRender.com/gfmro4y.

Previous work has established the mammalian target of rapamycin (mTOR) as a key coordinator of cellular growth, metabolism, survival, and proliferation in both physiological and pathological contexts [4]. Furthermore, mTOR has been extensively demonstrated to regulate both innate and adaptive immune responses [5,6]. Through the activity of its two primary complexes, mTOR complex 1 (mTORC1) and complex 2 (mTORC2), mTOR coordinates metabolic reprogramming and transcriptional responses that influence immune cell phenotype and function [7,8,9]. In microglia, mTOR has been associated with the regulation of inflammatory states including aging and neurodegeneration [10]. Furthermore, dysregulation of mTOR activity is implicated in numerous neuroinflammatory conditions, where alterations in microglial phenotypes can impact pathological progression [11,12,13]. This review will summarize our current understanding of the role of mTOR in microglial phenotypic polarization as well as the potential downstream mechanisms by which mTOR may exhibit these effects, summarized in Figure 1. By integrating these findings, this review aims to delineate how mTOR may function as a regulator of microglial state transitions and highlight its potential as a therapeutic target for neuroinflammatory conditions.

## 2. Microglial Polarization

Microglia function to perform immune surveillance, provide trophic support, and maintain homeostasis within the central nervous system (CNS) [1]. Similarly to macrophages, microglial phenotypes have been historically categorized into resting (M0), classically activated (M1), and alternatively activated (M2) [1,3]. While this classification provided an early conceptual foundation for microglial phenotypes, it is now widely recognized as an oversimplification [14]. Advances in single-cell transcriptomics, spatial profiling, and multi-omic analyses have revealed that microglia adopt a broad spectrum of highly dynamic and context-dependent states, necessitating a shift toward continuum-based models of phenotypic identity [2].

Under standard physiological conditions, microglia predominantly present in a homeostatic or surveilling state. Morphologically, these cells exhibit a small soma size with highly motile, ramified processes that constantly survey the brain parenchyma. This constant surveillance enables microglia to detect subtle changes in the microenvironment, such as pathogen- or damage-associated molecular patterns (PAMPs or DAMPs) and rapidly respond to them. In addition to their immune sentinel function, homeostatic microglia support the CNS through clearance of apoptotic cellular debris, synaptic pruning, trophic support of neurons and glia, as well as regulation of neuronal excitability. At the molecular level, this state is commonly associated with the expression of canonical markers purinergic receptor P2Y (P2RY12), transmembrane protein 119 (TMEM119), tyrosine-protein kinase MER (MerTK), and C-X3-C motif chemokine receptor 1 (CX3CR1) [15,16,17].

Importantly, the definition of a homeostatic microglial state is not universally fixed. For example, during embryonic and early postnatal development, microglia exhibit pronounced heterogeneity, adopting specialized phenotypes that support neurodevelopmental processes such as synapse refinement and circuit formation [18,19,20]. These findings underscore the importance of contextualizing microglial states through developmental stage, anatomical location, and the local signaling environment, rather than relying on static marker-based definitions [2].

Upon detection of inflammatory stimuli, microglia undergo phenotypic shifts toward activated states that can range from neuroprotective to neurotoxic [21]. Recognition of PAMPs or DAMPs through pattern recognition receptors (PRRs), including Toll-like receptors (TLRs) or NOD-like receptors (NLRs), initiates intracellular signaling cascades that drive various transcriptional and functional changes [21,22,23,24]. Classically described pro-inflammatory activation states are characterized by secretion of pro-inflammatory mediators including interleukin-1 beta (IL-1β), interleukin-6 (IL-6), tumor necrosis factor-alpha (TNF-α), chemokines such as C-X-C motif chemokine ligand 10 (CXCL10), and reactive oxygen or nitrogen species [25,26,27]. Furthermore, at the molecular level, pro-inflammatory microglia will often downregulate their expression of homeostatic markers and upregulate markers such as human leukocyte antigen-DR subtype (HLA-DR), inducible nitric oxide synthase (iNOS), and cyclooxygenase-2 (COX2) [25,28,29]. These pro-inflammatory states also classically exhibit an increased soma size, retraction of branches, and increased phagocytic activity [30,31,32]. These responses are critical for pathogen clearance and initiation of immune defense; however, inflammatory states become detrimental if sustained or dysregulated, thus contributing to chronic neuroinflammation and neuronal damage [33].

In contrast, microglia can also adopt neuroprotective or anti-inflammatory phenotypes that promote the resolution of inflammation and tissue repair. These states are classically grouped under an “M2-like” classification, though this does not entirely capture their diversity. Anti-inflammatory microglia are typically associated with the secretion of anti-inflammatory cytokines including interleukin-4 (IL-4), interleukin-10 (IL-10), and transforming growth factor-beta (TGF-β), as well as expression of arginase 1 (ARG1), suppressor of cytokine signaling 3 (SOCS3), and CD206 [25,34,35,36,37]. The induction of these phenotypes is highly dependent on environmental cues including cytokines, growth factors, and metabolic signals within the CNS microenvironment [38]. Emerging evidence suggests that these phenotypic programs can coexist with or transition into other states dependent on the balance of local signals [39].

However, even within these so-called pro- or anti-inflammatory states, microglial responses are highly heterogeneous and shaped by many factors including stimulus type, duration, metabolic state, and interactions with neighboring cells [39,40]. Recent studies highlight the existence of specialized subtypes or states of microglia tied to disease, aging, and development. These include disease-associated microglia, proliferative-associated microglia, white matter-associated microglia, each with distinct transcriptional signatures [2]. Together, these observations reinforce the concept that microglial polarization is best understood as a multidimensional, plastic continuum rather than a strict classification system.

## 3. The Mammalian Target of Rapamycin

The mammalian target of rapamycin (mTOR) is a highly conserved serine–threonine protein kinase that plays a central role in the regulation of cell growth, metabolism, survival, and homeostasis [4]. mTOR belongs to the phosphoinositide 3-kinase (PI3K)-related family and functions as the catalytic core of two distinct multiunit complexes, mTOR complex 1 (mTORC1) and 2 (mTORC2) [41]. Although both complexes share the catalytic mTOR subunit, the mammalian lethal with sec-13 protein 8 (mLST8), the DEP domain containing mTOR-interacting protein (DEPTOR), and the TELO2-interacting protein 1/telomerase maintenance 2 (TTI1/TEL2) chaperone system, they differ in accessory components, upstream regulatory input, downstream substrates, and sensitivity to rapamycin [42,43,44]. Rapamycin, the most prevalent canonical inhibitor of mTOR, forms a complex with mTOR’s FK506-binding protein 12 (FKBP12) and selectively inhibits mTORC1 by allosterically impairing substrate access, whereas mTORC2 is considered relatively insensitive to acute rapamycin exposure [45,46].

### 3.1. mTORC1

mTORC1, composed of mTOR, regulatory associated protein of mTOR (RAPTOR), proline-rich AKT substrate of 40 kDa (PRAS40), mLST8, and DEPTOR, serves as the primary regulator of cellular anabolism and metabolism [47,48,49]. It drives protein synthesis through phosphorylation of the eukaryotic translation initiation factor 4E (eIF4E)-binding proteins (4E-BPs), enhances lipid synthesis via sterol regulatory element-binding proteins 1 and 2 (SREBP1/2), and supports glycolytic reprogramming through hypoxia-inducible factor 1-alpha (HIF-1α) activation [50,51,52,53]. Additionally, mTORC1 negatively regulates autophagy through inhibition of Unc-51-like-kinase (ULK1) and limits lysosome biogenesis by phosphorylation of transcription factor EB (TFEB) [54,55,56].

Canonically, mTORC1 is activated by amino acids, particularly leucine and arginine, as well as growth factors. Growth factors such as insulin and insulin growth factor-1 (IGF-1) stimulate the PI3K-AKT and RAS-ERK pathways, leading to phosphorylation and inhibition of the tuberous sclerosis complex 1/2 (TSC1/2) [48]. Protein kinase B (AKT), extracellular-signal regulated kinase 1/2 (ERK1/2), and ribosomal S6 kinase (RSK1) directly phosphorylate the TSC1/2 heterodimer, relieving its inhibitory effect on Ras homolog enriched in the brain (RHEB) GTPase [57,58,59,60,61]. As a GTPase-activating protein for RHEB, TSC1/2 maintains RHEB in its inactive state; when RHEB is GTP-loaded, it directly binds and activates mTORC1 [62,63]. Independently, amino acid stimulation engages RAG GTPases through GATOR1/2 signaling to recruit mTORC1 via its RAPTOR subunit to the lysosomal surface, where GTP-bound RHEB activates it [47,64]. In addition to these canonical inputs, mTORC1 is also regulated by cellular stress, particularly hypoxia or low energy states, which converge on TSC1/2 and related inhibitory pathways to restrain its activity [63].

### 3.2. mTORC2

mTORC2 is composed of mTOR, rapamycin-insensitive companion of mTOR (RICTOR), mammalian stress-activated mitogen-activated protein kinase (MAPK)-interacting protein 1 (mSIN1), protein observed with Rictor 1 and 2 (PROTOR1/2), and mLST8 [49,65,66]. It primarily regulates cell survival, polarity, and cytoskeletal dynamics [67]. Compared to mTORC1, the upstream regulators and downstream effectors of mTORC2 are far less defined. Growth factors such as insulin and IGF-1 activate PI3K to generate phosphatidylinositol (3,4,5)-triphosphate (PIP3), recruiting mTORC2 via the pleckstrin homology (PH) domain of mSIN1 and relieves autoinhibition to promote mTORC2 activation at the plasma membrane [68]. mTORC2 is also regulated by mSIN1 phosphorylation, although the functional consequences of mSIN1 phosphorylation appear context-dependent. One study reported that S6K1- or AKT-mediated phosphorylation of mSIN1 at Thr86 and Thr398 disrupts mTORC2 integrity and suppresses downstream AKT signaling [69]. In contrast, AKT-mediated phosphorylation of Thr86 alone has been associated with enhanced mTORC2 activity and a positive AKT-mTORC2 feedback loop [70]. Therefore, the functional consequence of mSIN1 phosphorylation is likely dependent on upstream kinase activity and residue-specific phosphorylation.

The major downstream targets of mTORC2 are members of the AGC kinase family, including AKT, protein kinase C (PKC) isoforms, and serum- and glucocorticoid-induced kinase 1 (SGK1). mTORC2 phosphorylates AKT to enhance metabolic and survival signaling [71]. mTORC2 also promotes the maturation and stability of multiple PKC isoforms such as PKC alpha/zeta (PKCα/ζ) and supports SGK1 activation [65,66,72]. Through these kinase effectors, mTORC2 further modulates Rho-family GTPase signaling including RhoA- and Rac1-dependent pathways to influence actin remodeling, cell migration, ion transport, and cell survival [73]. Overall, mTORC2 coordinates structural and metabolic responses that complement the anabolic functions of mTORC1.

## 4. Involvement of mTOR in Microglial Polarization

mTOR has been previously implicated in microglial polarization as a regulator of both the directionality and intensity of microglial phenotypic states. Activation of mTORC1 is generally considered to promote a more neurotoxic microglial phenotype, whereas inhibition of this pathway reduces inflammatory outputs. In contrast, though far less studied than mTORC1, mTORC2 is thought to foster a more anti-inflammatory microglial phenotype. This is summarized in Table 1.

### 4.1. mTORC1: A Context-Dependent Amplifier of Microglial Polarization

Much of the evidence for mTOR-dependent microglial polarization stems from disease models, particularly those in aging and neurodegeneration. In aged mice, microglia exhibited hyperactivation of mTORC1, leading to an amplified microglial inflammatory response to inflammatory stimuli [10]. Interestingly, reduction in mTORC1 activity via *Rheb1* knockout increased the expression of priming or inflammatory genes at the mRNA level. Yet, inflammatory protein production was reduced, indicating uncoupling of transcriptional priming and translational output [10]. A similar mTORC1-dependent activation pattern has been observed in a mouse model of Niemann-Pick type C disease, where microglia exhibited hyperactivation of mTORC1 [74]. This hyperactive mTORC1 was accompanied by increased proliferation, phagocytic activity, ameboid morphology, and elevated *Hif1a* expression; inhibition of mTORC1 reduced these inflammatory features [74]. In mouse and rat models of ischemia, where microglia were exposed to hypoxic conditions, mTORC1 was elevated, resulting in production of pro-inflammatory cytokines and chemokines as well as metabolic reprogramming, which was suppressed when mTORC1 signaling was inhibited or attenuated [75,76]. Microglial mTORC1 was also upregulated in response to disease non-specific stimuli, such as lipopolysaccharide (LPS) and adenosine triphosphate (ATP), which was reduced by rapamycin [77].

Importantly, the role of mTORC1 in microglial polarization is highly context-dependent. In some inflammatory settings, rapamycin reduced microglial iNOS and COX2 expression, consistent with suppression of a pro-inflammatory phenotype [78,79]. However, other studies have reported enhanced microglial COX2 production when mTORC1 activity was altered via rapamycin treatment [80]. This variability suggests that mTORC1 shapes microglial phenotype according to the nature of upstream signaling and disease context. A striking example of this is in glioma, where both primary rat microglia and immortalized human microglia have shown hyperactivation of mTORC1 in response to the tumor microenvironment and adopt a more anti-inflammatory phenotype [7,81]. In this setting, inhibition of mTORC1 shifted tumor-associated microglia toward a pro-inflammatory phenotype and suppressed acquisition of a neuroprotective state [7,81,82]. Because anti-inflammatory microglia can support tumor growth, mTORC1 inhibition may be an advantageous therapeutic strategy in glioma [83]. Together, these studies underscore mTORC1 as a central but highly context-dependent regulator of microglial polarization, with effects that vary according to the nature of the inflammatory stimulus.

### 4.2. mTORC2: A Modulator of Anti-Inflammatory and M2-like Responses

Compared to mTORC1, the role of mTORC2 in microglial polarization has been investigated far less directly. Much of the current framework is inferred from a limited number of macrophage studies; however, one study has found that activation of a C-C chemokine receptor type 4 (CCR4)/mTORC2 signaling axis in primary rat microglia increased expression of the neuroprotective markers CD201, mannose receptor C-type 1 (MRC1), and ARG1, and reduced neuronal apoptosis [84]. In macrophages, mTORC2 signaling is understood to be essential for M2 polarization and is a therapeutic target for vascular inflammation and atherosclerosis [9,85]. Interestingly, inhibition of mTORC2 in macrophages appears to promote the M1 phenotype in numerous cancers, including melanoma and colorectal cancer [86,87]. These observations support a model in which mTORC2 generally promotes anti-inflammatory polarization in microglia or macrophages; however, further microglia-specific studies are required to better define its function.

## 5. Microglial Receptors and Upstream Inputs to mTOR Signaling

Microglia integrate extracellular cues through numerous receptors that regulate survival, metabolism, phagocytosis, and inflammatory signaling. Many of these receptors converge on pathways that may influence mTORC1 and/or mTORC2 signaling including PI3K-AKT, RAS-ERK, AMP-activated protein kinase (AMPK), and NFκB [88,89,90,91]. Through these signaling networks, receptor-dependent regulation of mTOR signaling may represent an important mechanism through which microglia utilize environmental cues to shift into distinct metabolic and functional response programs.

### 5.1. Triggering Receptor Expressed on Myeloid Cells 2

Triggering receptor expressed on myeloid cells 2 (TREM2) is a microglial receptor that recognizes a broad range of lipid- and damage-associated ligands through the adaptor proteins DNAX-activating protein 12 (DAP12) and DNAX-activating protein 10 (DAP10) [92,93]. TREM2 activation leads to DAP12 phosphorylation via Src family kinases to faciliate recruitment of spleen tyrosine kinase (SYK) [93]. This initiates downstream signaling cascades that may regulate mTOR activity including PI3K, PKC, and ERK [94].

TREM2 has been specifically linked to PI3K/AKT/mTOR-dependent regulation of microglial metabolic fitness, autophagy, and survival [95,96]. In a mouse model of Alzheimer’s disease, loss of TREM2 reduced mTOR-pathway activity and impaired metabolic fitness and cell survival [95]. Additionally, in models of Alzheimer’s and Parkinson’s disease, TREM2-mTOR signaling contributed to microglial responses to protein aggregation. TREM2 deficiency reduced microglial clustering and clearance of amyloid-β plaques, whereas deletion of the mTOR inhibitor *Tsc1* increased *Trem2* expression and enhanced amyloid-β clearance [93,95,97,98,99]. These observations position TREM2 as an important link between microglial damage sensing and mTOR-dependent microglial programming.

### 5.2. Pattern-Recognition Receptors

Pattern-recognition receptors (PRRs) allow microglia to detect PAMPs and DAMPs generated during infection, inflammation, or injury. TLRs, particularly TLR2 and TLR4, are the most comprehensively studied microglial PRRs. When TLRs detect a PAMP or DAMP, they signal through adaptor proteins such as myeloid differentiation primary response protein 88 (MyD88) and, for certain TLRs, Toll/interleukin-1 receptor domain-containing adaptor protein inducing interferon-beta (TRIF). These pathways activate NFκB, MAPK signaling, and interferon-associated transcriptional responses to regulate expression of inflammatory effectors.

The literature focusing on the TLR/mTOR signaling axis in microglia and macrophages is limited; however, TLR signaling has also been shown to mediate PI3K/AKT/mTOR signaling in other innate immune cells, thus coupling innate immune recognition to metabolic reprogramming and inflammatory outputs [100]. Studies thus far in microglia have shown that TLR5 activation leads to PI3K/AKT/mTORC1 signaling, and that the TLR4/MyD88-mTOR signaling pathway increases pro-inflammatory microglial signaling [100,101].

## 6. Downstream Effectors of mTOR Contributing to Microglial Polarization

While the overarching role of mTOR in immune cell metabolic reprogramming is well-established, the specific downstream effectors mediating these pathways in microglia remain an emerging area of investigation. However, clear links between phenotypic polarization and mTOR signaling have been explained in the macrophage literature [102]. Consequently, drawing upon this foundational knowledge and combining it with emerging findings in microglia provides a framework for exploring how mTOR-dependent signals may sculpt microglial polarization states.

### 6.1. mTORC1: Metabolic and Translational Control of Inflammatory Responses

mTORC1 is a critical driver of the metabolic reprogramming required for microglial phenotypic polarization [103]. By promoting the stabilization of HIF-1α, mTORC1 shifts cellular metabolism toward aerobic glycolysis, providing the energetic substrate necessary for inflammatory responses [104,105]. Additionally, the mTORC1/HIF-1α signaling axis is an essential component of microglial innate immune memory, where inhibition of mTORC1 with rapamycin inhibits microglial innate immune memory and reduces pro-inflammatory cytokine and chemokine secretion [106].

Beyond metabolism, mTORC1 modulates transcriptional and translational machinery to amplify inflammatory outputs. In both microglia and macrophages, mTORC1 activation has been reported to facilitate nuclear factor kappa-light-chain-enhancer of activated B cells (NFκB) p65 phosphorylation through NFκB inhibitor alpha (IκB-α) degradation, and to promote signal transducer and activator of transcription 3 (STAT3) activation via TLR-dependent mechanisms [107,108]. Aberrant STAT3 activation has been shown to lead to microglial neurotoxicity in numerous disease models [109,110,111]. In conjunction, activation of mTORC1 in macrophages may inhibit the anti-inflammatory adaptor protein SOCS3, a negative regulator of STAT3, thus reinforcing pro-inflammatory signaling [112]. Simultaneously, microglial mTORC1 enhanced the translation of inflammatory cytokines and other mediators through the 4EBP1/eIF4E-dependent pathway which relies on cap-dependent translation [10]. In microglia, mTORC1 can also act upstream of the P38 mitogen-activated protein kinase pathway through phosphorylation of mitogen-activated protein kinase kinase 4 (MKK4), which directly mediates the production of the inflammatory cytokines including IL-1β, TNFα, and IL-6 [113,114]. Together, these pathways position mTORC1 as an amplifier of microglial neurotoxic polarization by coordinating metabolic reprogramming, inflammatory gene expression, and cytokine translation.

### 6.2. mTORC2: A Regulator of Anti-Inflammatory and Cytoskeletal Responses

In contrast to the predominantly pro-inflammatory role of mTORC1, mTORC2 supports adaptive and restorative functions, acting as a regulator of cellular homeostasis and cytoskeletal organization. A key downstream effector of mTORC2 is interferon regulatory factor 4 (IRF4) in parallel with signal transducer and activator of transcription 6 (STAT6), which supports oxidative phosphorylation and fatty acid oxidation; these metabolic processes are essential for the M2 activation of macrophages [99]. Furthermore, mTORC2 activation inhibits the forkhead box protein O1 (FOXO1) transcription factor in macrophages, attenuating IL-1β secretion [85].

mTORC2 also modulates microglial structure and function through its control of AGC kinase pathways and the cytoskeleton. Specifically, mTORC2 is responsible for the activation of PKCα, which governs actin dynamics, cellular mobility, and phagocytic activity [115,116]. Similarly, mTORC2-mediated activation of SGK1 acts as a checkpoint for microglial homeostasis, where deficiency of SGK1 in microglia has been shown to promote uncontrolled proliferation, excessive inflammatory cytokine release, as well as an ameboid, reactive morphology [117]. Thus, while mTORC1 acts as an amplifier of inflammatory metabolic and transcriptional programs, mTORC2 provides regulation of tissue-repair phenotypes and cytoskeletal plasticity.

## 7. Therapeutic Implications of Targeting mTOR in Microglia

Pharmacological targeting of mTOR represents a potential strategy for modulating microglial neuroinflammatory responses; however, mTOR-targeting drugs should not be uniformly viewed as pro- or anti-inflammatory strategies. Their consequences on microglial state are shaped by inhibitor class, degree and duration of pathway inhibition, dose, duration, blood–brain barrier (BBB) penetrance, and disease context. In particular, while suppression of inflammatory mediators may be beneficial in settings characterized by mTORC1 hyperactivation, mTOR activity may support important microglial functions such as phagocytosis or disease clearance. Accordingly, therapeutic strategies should seek to improve balance of microglial functions rather than indiscriminately suppressing microglial activation.

### 7.1. Allosteric Inhibitors of mTOR

The most clinically established mTOR inhibitors are sirolimus (rapamycin) and its analogs, or rapalogs, including everolimus (RAD001) and temsirolimus [118]. Mechanistically, these drugs form a complex with FKBP12 and allosterically inhibit mTORC1 by restricting substrate access to the mTOR kinase complex [119]. While rapamycin-class compounds are classically considered to be mTORC1-selective, prolonged treatment may impair mTORC2 assembly or signaling [120].

In several preclinical microglia models, rapamycin-class compounds have attenuated select mTORC1-associated inflammatory outputs. Both rapamycin and everolimus have been reported to reduce iNOS expression, nitrite production, and cytokine-associated signaling in inflammatory microglia models [7,78,106,121]. Rapamycin specifically has been shown to suppress immune memory in microglia, resulting in reduced pro-inflammatory cytokine production [106]. In LPS- and kainic acid-induced neuroinflammatory paradigms, everolimus displayed greater suppression of iNOS expression and nitrite production than rapamycin, suggesting that there are differences in pharmacologic properties and signaling effects [121]. Additionally, everolimus also reduced microglial activation in rats following kainic acid-induced seizures, leading to reduced neuronal apoptosis and seizure susceptibility and intensity [122]. In a mouse model of vascular dementia, use of everolimus was shown to improve symptoms of vascular cognitive impairment by restoring a balance of microglial states [110].

However, allosteric mTOR inhibition does not uniformly suppress pro-inflammatory microglial states or lead to beneficial outcomes. In primary rat microglia exposed to glioma conditioned medium, rapamycin and everolimus both increased iNOS and nitric oxide production while reducing neuroprotective markers arginase-1 and IL-10 expression [7]. This shift away from a pro-tumor microglial state suggests that increased inflammatory output may be therapeutically advantageous for glioma. Similarly, rapamycin increased IL-6 secretion in IL-1β-stimulated HMC3 microglia, demonstrating that mTORC1 inhibition may selectively enhance inflammatory mediators depending on the stimulus [123]. Thus, rapalogs are not uniformly anti-inflammatory microglial mediators of microglial states, but rather they alter the balance of microglial programs in a context-specific manner.

Attenuation of mTORC1-associated signaling may be beneficial in contexts characterized by sustained mTORC1 hyperactivation; however, mTORC1 signaling can also contribute to overall microglial metabolic fitness, lysosomal function, proliferation, and phagocytic responses [74,95]. Thus, sustained or nonselective mTOR suppression may impair beneficial microglial programs. For example, prolonged rapamycin treatment in a mouse model of Alzheimer’s disease was associated with reduced microglial TREM2 expression, diminished amyloid-β uptake and clearance, and overall exacerbation of amyloid-related pathology [97]. Similarly, rapamycin reduced the phagocytic-associated microglia marker CD11C+ and increased amyloid plaque load in Alzheimer’s mice [124]. These studies illustrate that reduced inflammatory activation via mTORC1 suppression does not necessarily predict therapeutic benefit, particularly in neurodegenerative disease in which microglia may support debris clearance.

Collectively, preclinical evidence supports rapamycin-class compounds as modulators, rather than uniform suppressors of microglial responses. In disorders where mTORC1 hyperactivation has been identified, mTORC1 inhibition may reduce maladaptive inflammatory signaling. Conversely, chronic inhibition of mTORC1 may impair necessary microglial functions via impairment of microglial metabolic fitness, lysosomal function, and phagocytic clearance. Future studies should define treatment windows and functional outcomes in disease-specific contexts.

### 7.2. Adenosine Triphosphate (ATP)-Competitive mTOR Inhibitors

ATP-competitive mTOR inhibitors are the second-generation of mTOR-targeting agents that target the ATP-binding site or orthosteric site of the mTOR kinase domain [125]. In contrast to rapamycin and rapalogs, which allosterically and incompletely inhibit mTORC1 outputs, ATP-competitive inhibitors generally suppress both mTORC1 and mTORC2 [125,126]. Their broader activity may provide an advantage in disorders in which rapamycin-resistant outputs contribute to pathology [127]. This class includes Torin1, Torin2, AZD8055, sapanisertib, and vistusertib (AZD2014) [128,129]. These agents may provide broader suppression of mTOR-driven inflammatory pathways compared to rapalogs; however, dual mTORC1/mTORC2 inhibition could simultaneously suppress mTORC2-dependent AKT, PKC isoforms, or SGK1 signaling, which support cell survival, metabolism, and cytoskeletal organization [117,130]. Consequently, a catalytic inhibitor may reduce pathological microglial activation in one disease setting but impair reparative or phagocytic functions in another. This issue is of importance considering the limited evidence defining mTORC2 function in microglia.

In a subarachnoid hemorrhage model, both rapamycin and AZD8055 reduced microglial activation and shifted microglia toward a neuroprotective phenotype, and were associated with recued neuronal apoptosis, decreased brain edema, and improved BBB integrity [130]. Thus, inhibition may improve injury-associated outcomes in contexts where microglial response programs worsen pathology. In contrast, a study comparing sapanisertib and in the context of glioma suggests that sapanisertib may polarize microglia toward a pro-tumor phenotype, whereas rapamycin appeared to polarize microglia toward a more inflammatory, anti-tumor phenotype [7]. This emphasizes that catalytic mTOR inhibitors are not necessarily more effective or potent versions of rapamycin, but that their activity may produce different consequences for microglial state regulation, particularly in glioma.

Torin1 provides additional evidence of the mechanistic difference in ATP-competitive inhibitors from rapamycin. In aged mouse microglia, Torin1 decreased 4E-BP1 phosphorylation more effectively than rapamycin and decreased inflammatory cytokine production despite increased transcript abundance in aged mouse microglia [10]. Thus, catalytic mTOR inhibition may better suppress the mTORC1-4E-BP1-eIF4E translational axis than traditional rapamycin class compounds. Torin1 has also been reported to enhance microglial clearance of neuronal alpha-synuclein through autophagy-dependent degradation [131].

Taken together, these findings suggest that ATP-competitive mTOR inhibitors may provide more complete suppression of mTOR-dependent translation of inflammatory mediators than rapalogs while also modulating autophagic and proteostatic pathways. Still, broad suppression of both mTOR complexes may create substantial risk of suppressing potential protective or restorative mTORC2-associated functions.

### 7.3. CNS-Restricted Inhibition of mTOR

A major translational constraint of current mTOR-targeting drugs is off-target tissue toxicity. Systemic inhibition of mTOR can produce dose-limiting adverse effects, including immunosuppression and metabolic disorders [132]. This may be particularly relevant for neuroinflammatory disease where long-term treatment may be required, and where patients may have age-related risks for metabolic comorbidities or vulnerability to infection [133,134]. Considering these risks, CNS-restricted mTOR inhibition is an emerging strategy of interest to preserve efficacy within the brain while minimizing toxicity in the periphery. An approach to this utilizes a combination of RapaLink-1 and RapaBlock, an mTOR inhibitor and a ligand of FKBP12, respectively [135]. Rapalink-1 is brain-penetrant and introduces a third-generation of mTOR inhibitors that act as bitopic ligand by simultaneously interacting with the orthosteric and allosteric binding pockets of mTOR [136]. In contrast, RapaBlock is brain-impermeable and occupies the FKBP12 binding site [135]. Thus, this combination limits the formation of the RapaLink-1-FKPB12 mTOR inhibition complex in peripheral tissues while permitting mTOR inhibition in the CNS. In mouse models of glioblastoma, this approach has been shown to retain intracerebral mTOR inhibition and anti-tumor efficacy while reducing common on-target peripheral side effects such as weight loss [135].

While this strategy remains in very early stages of preclinical development, it shows promise for tissue-selective mTOR pharmacology. Future approaches should incorporate assessment of brain distribution, cell-type-specific target engagement, and long-term consequences for the CNS as a whole.

## 8. Conclusions

Control of microglial phenotypic plasticity through mTOR signaling reveals a complex regulatory axis that coordinates metabolic reprogramming, translational control, and inflammatory responses within the CNS. The evidence reviewed here supports a model in which mTOR signaling contributes to microglial state regulation through coordinated effects on metabolism, protein translation, autophagy–lysosomal pathways, cytoskeletal organization, proliferation, and more. mTORC1 may be a context-dependent amplifier of pro-inflammatory or neurotoxic polarization, driving glycolytic metabolism, cytokine translation, and NFκB activation to sustain chronic inflammation in neuroinflammatory disorders. In contrast, mTORC2 may support anti-inflammatory, neuroprotective repair through AKT/PKC/SGK1-mediated cytoskeletal dynamics and oxidative phosphorylation; however, its role in microglia remains underexplored.

mTOR should not be considered a binary molecular switch determining whether microglia adopt uniformly neurotoxic or neuroprotective phenotypes; rather, mTORC1 and mTORC1 appear to bias microglial states along a dynamic continuum that is shaped by stimulus, disease stage, microenvironment, cellular metabolism, and the duration of pathway activation or inhibition. These divergent functions position mTOR as a regulator of microglial state transitions, where dysregulation may exacerbate pathological progression. Additionally, the interconnected nature of mTORC1 and mTORC2 suggests a need for proper balance of each complex to maintain cellular homeostasis.

As an emerging field, critical gaps in our understanding of mTOR’s regulation of microglial phenotypic states exist. It remains unclear how CNS-specific inflammatory cues, such as amyloid beta or myelin debris, stimulate upstream regulators of mTOR. Additionally, the temporal dynamics remain poorly defined, as acute versus chronic mTOR modulation may yield opposing phenotypic outcomes. Further exploration of these gaps may position mTOR as a therapeutic target for pathological conditions such as Alzheimer’s disease, stroke, and epilepsy.

## Figures and Tables

**Table 1:** Summary of reported associations between mTORC1/mTORC2 modulation on microglial or macrophage response programs across experimental contexts.

Experimental Context	Cell Model/System	mTORC Perturbation	Functional State
Aging	Wild-type mouse microglia	mTORC1 ↑	Primed for pro-inflammatory cytokine release [10]
Niemann-Pick type C disease	*Npc1^nmf164^* mouse brain	mTORC1 ↑	Increased proliferation, phagocytosis, ameboid morphology, and HIF-1α expression [74]
Ischemic stroke	Primary rat microglia and BV-2 microglia [75] Raptor^KO^ mouse brain [76]	mTORC1 ↑	Increased pro-inflammatory cytokine and chemokine production, glycolytic reprogramming [75,76]
LPS or ATP stimulation	Primary rat microglia	mTORC1 ↑	Increased pro-inflammatory mediator production [77]
Multiple sclerosis simulated by IL-1β, TNF-α, and IFN-γ treatment	Primary rat microglia	mTORC1 ↑	Increased pro-inflammatory mediator production [78]
Neuroinflammation modeled by catalase treatment	BV2 microglia	mTORC1 ↑	Increased pro-inflammatory mediator production [79]
LPS stimulation with inhibition of mTORC1	Primary rat microglia	mTORC1 ↓	Increased pro-inflammatory mediator production [80]
Glioblastoma	Primary rat microglia [7] C20 and HMC3 microglia [81] Human iPSC-derived microglia and primary mouse microglia [82] Primary human glioma tissue [83]	mTORC1 ↓	Increased pro-inflammatory cytokine production, tumor-associated microglial phenotype [7,81,82,83]
Subarachnoid hemorrhage with CCL17 stimulation	Primary rat microglia	mTORC2 ↑	CCL17 stimulates the CCR4/mTORC2 signaling axis, increased expression of neuroprotective markers CD201, MRC1, and *ARG1* in the context of subarachnoid hemorrhage [84]
Parasitic infection (helminth)	Bone-marrow derived mouse macrophages	mTORC2 ↓	Impaired M2 differentiation and helminth clearance [9]
Atherosclerosis	mTOR^Lyz2-Cre^ and Rictor^Lyz2-Cre^ mouse macrophages	mTORC2 ↓	Increased IL-1β transcription [85]
Melanoma	Rictor^Lyz2-Cre^ macrophages and RAW264.7 macrophages	mTORC2 ↓	Increased pro-inflammatory signaling and anti-tumor myeloid state characteristics [86]
Colorectal cancer	Rictor^Lyz2-Cre^ mouse macrophages	mTORC2 ↓	Reduced M2-like gene expression, increased M1-like gene expression [87]

“↑” and “↓” correlate to an increase or decrease in mTORC1 or mTORC2 activity.

## Data Availability

No new data were created or analyzed in this study. Data sharing is not applicable to this article.

## Abbreviations

The following abbreviations are used in this manuscript:

4E-BP	Eukaryotic translation initiation factor 4E-binding protein
AKT	Protein kinase B
AMPK	AMP-activated protein kinase
ARG1	Arginase 1
ATP	Adenosine triphosphate
CCR4	C-C chemokine receptor type 4
CNS	Central nervous system
COX2	Cyclooxygenase
CX3CR1	Central nervous system
CXCL10	C-X-C motif chemokine ligand 10
DAMP	Damage-associated molecular pattern
DAP10	DNAX-activating protein 10
DAP12	DNAX-activating protein 10
DEPTOR	DEP domain containing mTOR-interacting protein
eIF4E	Eukaryotic translation initiation factor 4E
ERK1/2	Extracellular-signal regulated kinase ½
FKBP12	FK506-binding protein 12
FOXO1	Forkhead box protein O1
HIF-1α	Hypoxia-inducible factor 1-alpha
HLA-DR	Human leukocyte antigen-DR subtype
IGF-1	Insulin growth factor-1
IL-10	Interleukin-10
IL-1β	Interleukin-1 beta
IL-4	Interleukin-4
IL-6	Interleukin-6
IFNγ	Interferon-gamma
iNOS	Inducible nitric oxide synthase
IRF4	Interferon regulatory factor 4
IκB-α	NFκB inhibitor alpha
LPS	Lipopolysaccharide
MAPK	Mitogen-activated protein kinase
MerTK	Tyrosine-protein kinase MER
MKK4	Mitogen-activated protein kinase kinase 4
mLST8	Mammalian lethal with sec-13 protein 8
MRC1	Mannose receptor C-type 1
mSIN1	Mammalian stress-activated MAP kinase-interacting protein 1
mTOR	Mammalian target of rapamycin
mTORC1	Mammalian target of rapamycin complex 1
mTORC2	Mammalian target of rapamycin complex
MyD88	Myeloid differentiation primary response protein 88
NFκB	Nuclear factor kappa-light-chain-enhancer of activated B cells
NLR	NOD-like receptor
P2RY12	Purinergic receptor P2Y
PAMP	Pathogen-associated molecular pattern
PI3K	Phosphoinositide 3-kinase
PIP3	Phosphatidylinositol (3,4,5)-triphosphate
PKCα/ζ	Protein kinase C alpha/zeta
PH	Pleckstrin homology
PRAS40	Proline-rich AKT substrate of 40 kDa
PROTOR1/2	Protein observed with Rictor 1 and 2
PRR	Pattern recognition receptor
RAPTOR	Regulator associated protein of mTOR
RHEB	Ras homolog enriched in the brain
RICTOR	Rapamycin-insensitive companion of mTOR
RSK1	Ribosomal S6 kinase
SREBP1/2	Sterol regulatory element-binding proteins 1 and 2
SGK1	Serum/glucocorticoid-regulated kinase 1
SOCS3	Suppressor of cytokine signaling 3
*STAT3*	Signal transducer and activator of transcription 3
*STAT6*	Signal transducer and activator of transcription 6
SYK	Spleen tyrosine kinase
TTI1	TELO2-interacting protein 1
TEL2	Telomere maintenance 2
*TFEB*	Transcription factor EB
TGF-β	Transforming growth factor-beta
TLR	Toll-like receptor
TMEM119	Transmembrane protein 119
TNF-α	Tumor necrosis factor alpha
TREM2	Triggering receptor expressed on myeloid cells 2
TRIF	Toll/interleukin-1 receptor domain-containing adaptor protein inducing interferon-beta
TSC1/2	Tuberous sclerosis complex ½
ULK1	Unc-51-like kinase
